# Supplementary material for: Circularly polarized electroluminescence from a single-crystal organic microcavity light-emitting diode based on photonic spin-orbit interactions
Source: Nat Commun. 2023 Jan 3;14:31. doi: 10.1038/s41467-022-35745-w (PMC9810703; doi:10.1038/s41467-022-35745-w)
Supplement: Supplementary file 1 — Supplementary Information [file 41467_2022_35745_MOESM1_ESM.pdf]

Supplementary Information:

Circularly polarized electroluminescence from a single-crystal organic microcavity light-emitting diode based on photonic spin-orbit interactions

Jichao Jia,[1,#] Xue Cao,[1,#] Xuekai Ma,[2] Jianbo De,[3] Jiannian Yao,[3] Stefan Schumacher,[2,4] Qing Liao,[1,*] Hongbing Fu[1,*]

[1]Beijing Key Laboratory for Optical Materials and Photonic Devices, Department of Chemistry, Capital Normal University, Beijing 100048, People's Republic of China

[2]Department of Physics and Center for Optoelectronics and Photonics Paderborn (CeOPP), Universität Paderborn, Warburger Strasse 100, 33098 Paderborn, Germany

[3]Institute of Molecule Plus, Tianjin University, and Collaborative Innovation Center of Chemical Science and Engineering (Tianjin), Tianjin 300072, P. R. China

[4]Wyant College of Optical Sciences, University of Arizona, Tucson, Arizona 85721, United States

[#]These authors contributed equally to this work

MATERIALS AND METHODS

1. Synthesis of 6M-DSB

The compound used in our work, 1,4-bis((E)-2,4-dimethylstyryl)-2,5-dimethylbenzene (6M-DSB), was synthesized (Figure S1) according to Horner-Wadsworth-Emmons reaction[1-2]. All starting materials were purchased from Sigma-Aldrich and used as received without further purification. The tetrahydrofuran (THF, HPLC grade) and hexane were purchased from Beijing Chemical Agent Ltd., China. Ultra-pure water with a resistance of 18.2 MΩ·cm^{-1} were used in all experiments, produced by Milli-Q apparatus (Millipore).

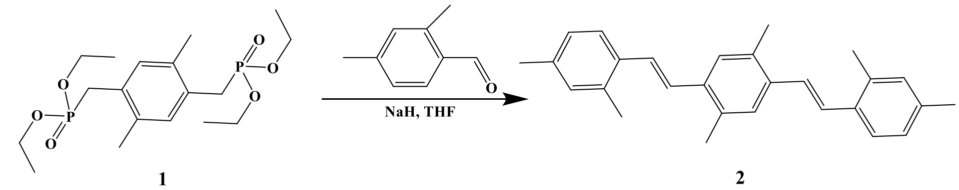

Figure S1. The synthesis of the 6M-DSB molecule.

A mixture of 2,5-dimethyl-1,4-xylene-bis(diethyl phosphonate) (1.06 g, 2.61 mmol) and the 2,4-dimethylbenzaldehyde (5.74 mmol) in tetrahydrofuran (THF) cooled in an ice bath was added 2 eq. NaH in small portions during a 30 min period. The reaction mixture was stirred at room temperature for 3 hours and poured into water. The phase was extracted with CH_2Cl_2. The pooled organic phases were washed with water, dried over anhydrous $MgSO_4$, filtered, and evaporated. The product was separated by flash chromatography on silica gel by means of CH_2Cl_2/petroleum ether (1:4). Finally a highly fluorescent powder was obtained as the title compound (573 mg) in 85% yield. The obtained 6M-DSB molecule was characterized by ^{1}H NMR (Figure S2).

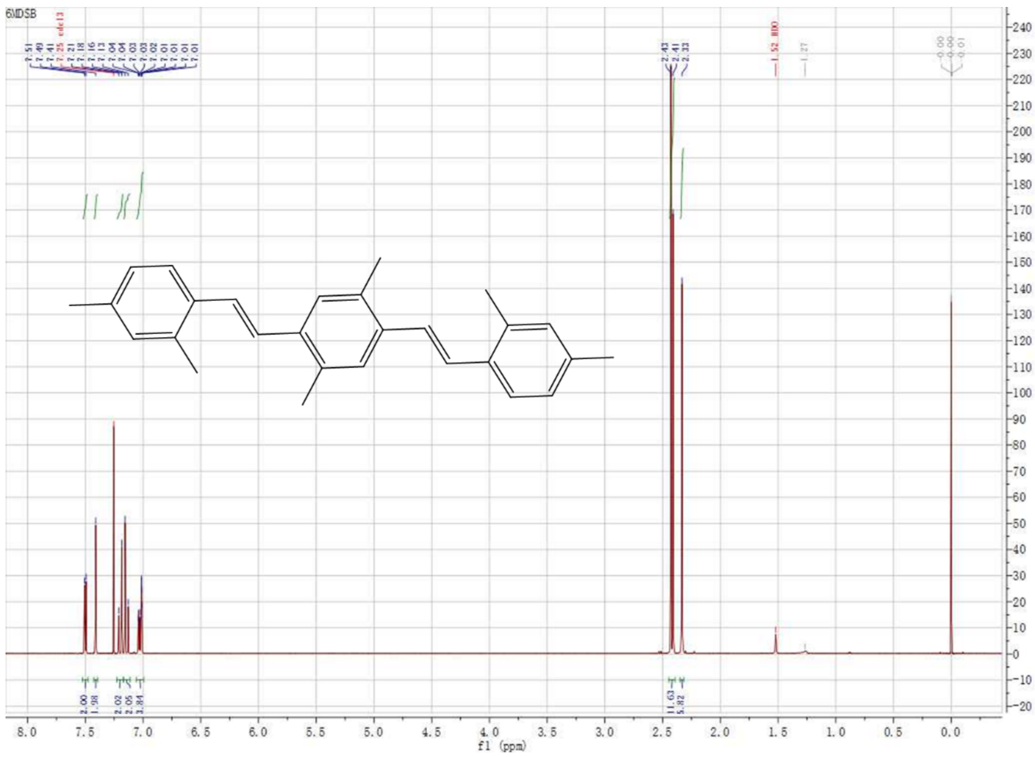

Figure S2. ^{1}H Nuclear magnetic resonance（NMR) spectrum of 6M-DSB.

1H NMR (600 MHz, Chloroform-d) δ 7.50 (d, J = 7.8 Hz, 2H), 7.41 (s, 2H), 7.20 (d, J = 15.9 Hz, 2H), 7.14 (d, J = 16 Hz, 2H), 7.06 – 6.99 (m, 4H), 2.42 (d, J = 11.1 Hz, 12H), 2.33 (s, 6H).

2. The preparation of 6M-DSB single crystals

In our experiment, 6M-DSB single crystals were fabricated using a facile physical vapor deposition (PVD) method. A quartz boat carrying 3 mg 6M-DSB was then placed in the center of a quartz tube which was inserted into a horizontal tube furnace. A continuous flow of cooling water inside the cover caps was used to achieve a temperature gradient over the entire length of the tube. To prevent oxidation of 6M-DSB, Ar was used as inert gas during the PVD process (flowrate: 100 sccm·min^{-1}). The pre-prepared hydrophobic substrates were placed on the downstream side of the argon flow for product collection and the furnace was heated to the

sublimation temperature of 6M-DSB (at temperature region of ~ 230 °C), upon which it was physically deposited onto the pre-prepared hydrophobic substrates for 3 hours.

The thickness of 6M-DSB crystal can be controlled by the PVD method in our experiments. For example, as presented in Figure S3, the crystal thickness obtained is about 380 nm when the deposition temperature is set to 150 °C and the flow rate of inert gas is set to 100 sccm·min^{-1}. As the deposition temperature is raised to 110 °C, the crystals with thickness of about 850 nm are obtained. When deposition temperature reaches 90 °C, the crystal thickness increases to about 1300 nm.

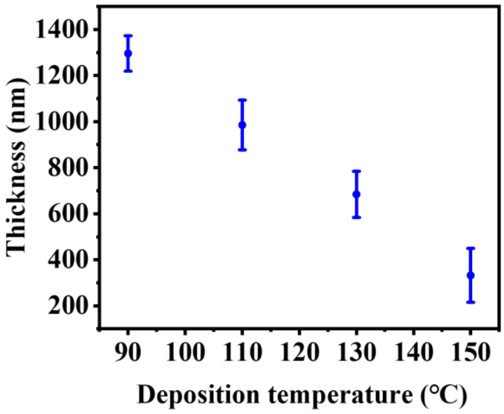

Figure S3. Dependence of the crystal thickness with standard deviation error bars on the deposition temperature.

3. The preparation of 6M-DSB single-crystal CP-OLEDs

Firstly, we use the metal vacuum deposition system (Amostrom Engineering 03493) to thermally evaporate a silver film with the thickness of 200 ± 5 nm (reflectivity R ≥ 99%) on a silicon wafer substrate, 6M-DSB crystals prepared by PVT are transferred to a silver substrate using a mechanical transfer method. The 6M-DSB crystals were uniformly dispersed on the silver film substrate. On this basis, the upper array of 10

nm cesium fluoride and 35 nm silver (R ≈ 50%) was prepared by the method of copper mesh mask to form microcavities. The 10 nm cesium fluoride layer is used to reduce the injection barrier between the electrode and the semiconductor layer to achieve good electron injection.

4. Structural and spectroscopic characterization

As-prepared 6M-DSB crystals were characterized by transmission electron microscopy (TEM, JEOL, JEM-2100) in which PVD grown crystals were mechanically transferred to a carbon-coated copper grid for testing. TEM measurement was performed at room temperature at an accelerating voltage of 100 kV. The X-ray diffraction (XRD, Japan Rigaku D/max-2500 rotation anode X-ray diffractometer, graphite monochromatized Cu K_α radiation (λ =1.5418 Å)) operated in the 2θ range from 3° to 30°, by using the samples on a cleaned glass slide.

The fluorescence micrograph, diffused reflection absorption and emission spectra were measured on Olympus IX71, HITACHI U-3900H, and HITACHI F-4600 spectrophotometers, respectively. The photoluminescence spectrum of the device was characterized by using a homemade optical microscope equipped with a 50 × 0.9 NA objective (Figure S4). A single selected device is excited on a two-dimensional (2D) movable table using a continuous laser focused at 405 nm to a 50-μm diameter spot. Spatially resolved PL spectra were collected underneath by using a 3D-movable objective and detected using a liquid-nitrogen cooled charge-coupled device (CCD).

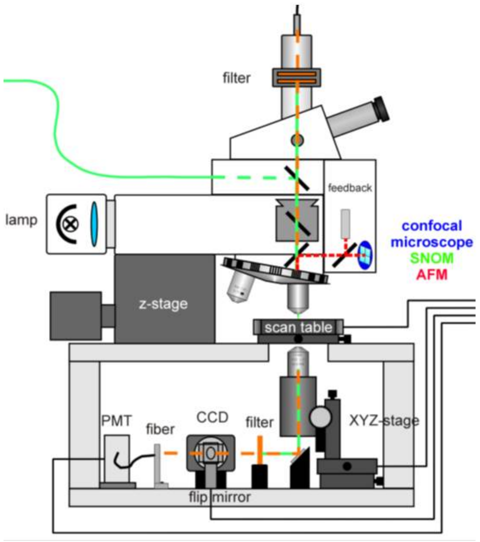

Figure S4. Schematic demonstration of the experimental setup for the optical characterization: the near-field scanning optical microscopy.

5. The angle-resolved spectroscopy characterization

The angle-resolved spectroscopy was performed at room temperature by the Fourier imaging using a 100× objective lens of a NA 0.95, corresponding to a range of collection angle of ±60° (Figure S5). The incident white light of the Halogen lamp with the wavelength of 400-700 nm is used to focus on the region to be measured. The k-space or angular distribution of the reflected light was located at the back focal plane of the objective lens. Lenses L1-L4 formed a confocal imaging system together with the objective lens, by which the k-space light distribution was first imaged at the right focal plane of L2 through the lens group of L1 and L2, and then further imaged, through the lens group of L3 and L4, at the right focal plane of L4 on the entrance slit of a spectrometer equipped with a liquid-nitrogen-cooled CCD. The use of four lenses here provided flexibility for adjusting the magnification of the final image and efficient light collection. Tomography by scanning the image (laterally shifting L4)

across the slit enabled obtaining spectrally resolved two-dimensional (2D) k-space images.

In order to investigate the polarization properties, we placed a linear polarizer, a half-wave plate and a quarter-wave plate in front of spectrometer to obtain the polarization state of each pixel of the k-space images in the horizontal-vertical (0° and 90°) and circular (σ+ and σ−) basis.

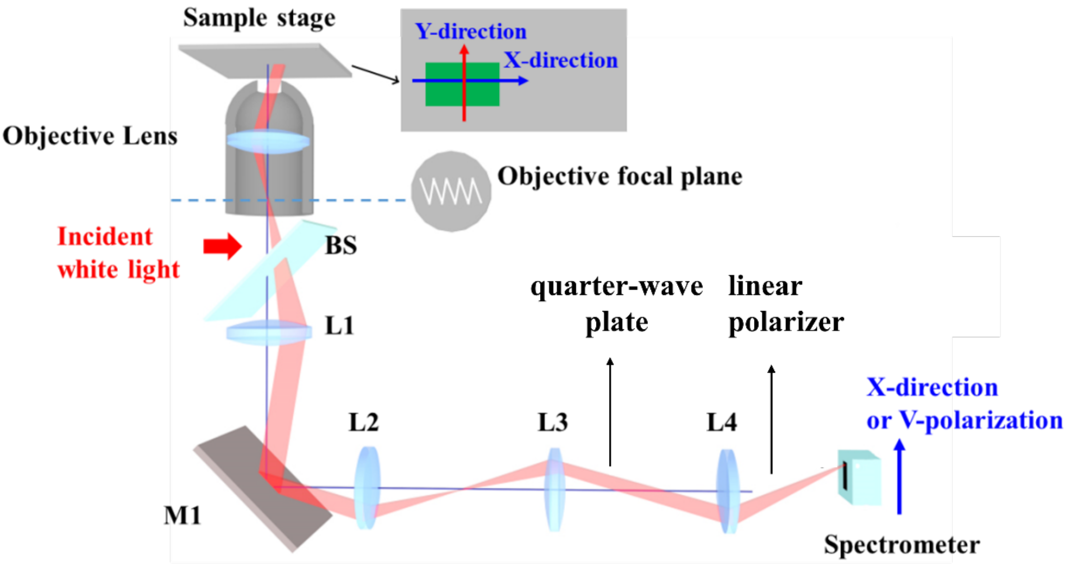

Figure S5. Experimental setup for the polarization-resolved PL and EL spectra. BS: beam splitter; L1-L4: lenses; M1: mirror. The red beam traces the optical path of the reflected light from the sample at a given angle.

PLQY of 6M-DSB single crystals

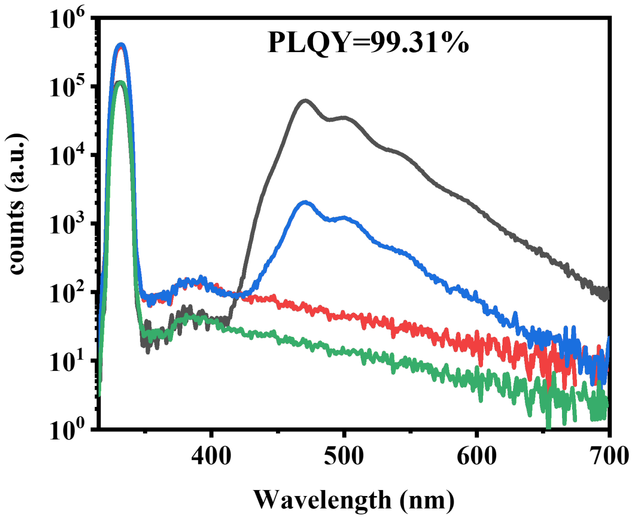

Figure S6. PLQY of 6M-DSB single crystals was measured through an absolute method by using an integration sphere in FLS-1000. At the excitation wavelength of 330 nm, PLQY of 6M-DSB crystals is determined to be 0.9931.

Morphology of 6M-DSB single crystals grown by the PVT method

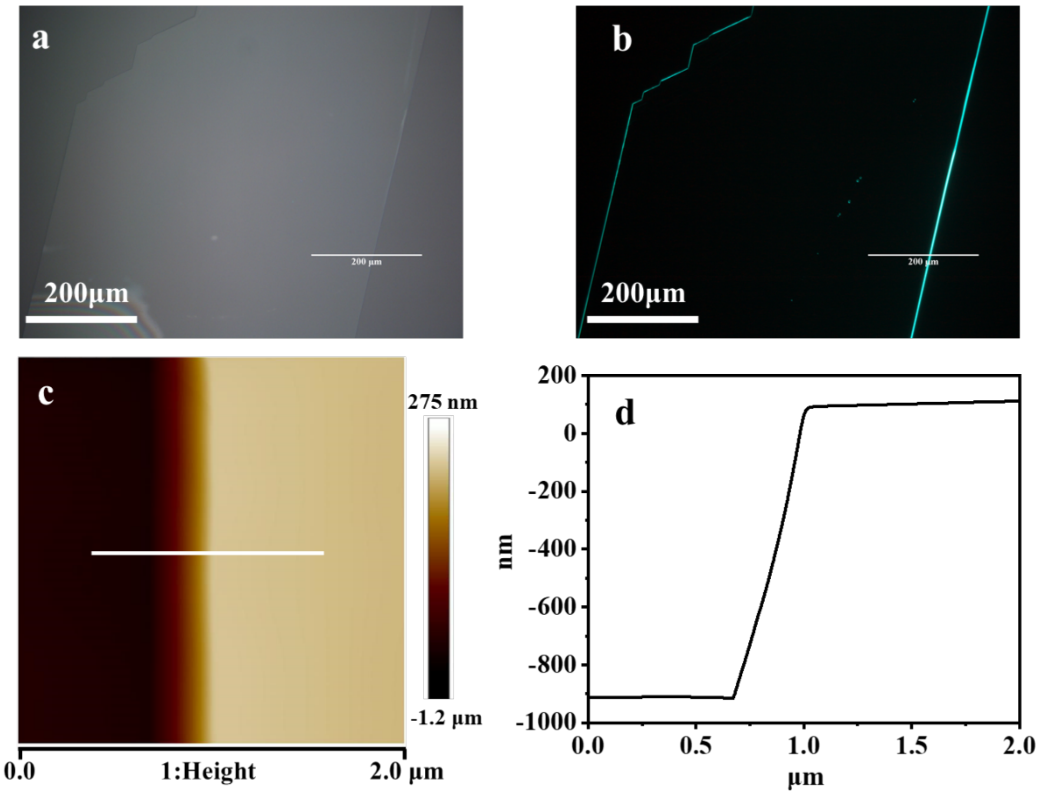

Figure S7. Microscopic bright field and fluorescence images of the 6M-DSB crystal. The crystal prepared by physical vapor deposition method are shown in (a) and (b). (c) The AFM image of the crystal used for device preparation. (d) The morphology curve along the white line in (c).

Characteristics of crystalline structure of 6M-DSB single crystals

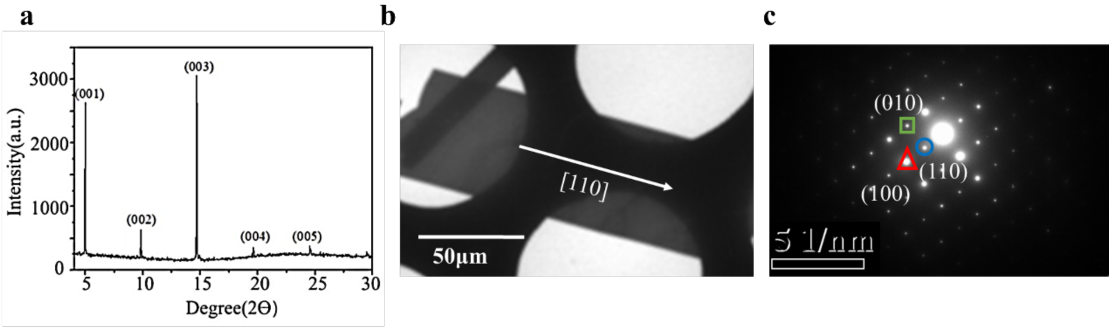

Figure S8. Characteristics of 6M-DSB single crystals. (a) XRD pattern of ensemble 6M-DSB microribbons filtered on the surface of an alumina membrane. (b) TEM image of a typical 6M-DSB microribbon. (c) SAED pattern of the microribbon in (b).

The XRD pattern was measured by a D/max 2400 X-ray diffractometer with Cu Kα radiation (λ = 1.54050 Å) operated in the 2θ range from 4° to 30°. According to the single-crystal data, the monoclinic crystal of 6M-DSB has the lattice parameters of a = 4.7533(10) Å, b = 5.9928(12) Å, c = 18.235(4) Å, α = 96.08(3)°, β = 96.46(3)°, and γ = 90.15(3)°. The XRD spectrum of microribbons shows a series of peaks corresponding to the (001) crystal plane with an interplanar spacing of 18.15 Å (Figure S8a). The observation of high-order diffraction peaks, such as (002)-(005), suggests that the crystal adopts a lamellar structure with the crystal (001) plane being parallel to the substrate. Figure S8c presents SEAD pattern recorded by directing the electron beam perpendicular to the flat surface of a single microribbon. The clearly observed SAED spots and its rectangular symmetry suggest that 6M-DSB microribbons are single crystals. The squared and triangled sets of SAED spots correspond to (020) and (100) crystal planes with d-spacing values of 6.10 and 4.76 Å, respectively, and the blue circled set of SAED spots are attributed to the (110) crystal

plane with a *d*-spacing value of 3.67 Å. Combining the XRD, SAED and TEM results (Figure S8b) together, the 6M-DSB ribbons grow along the [110] crystal direction, bound by (001) and (0-10) crystal planes on the top and bottom surfaces and (1-10) and (-110) crystal planes on the lateral surfaces.

Simulation of two cavity modes

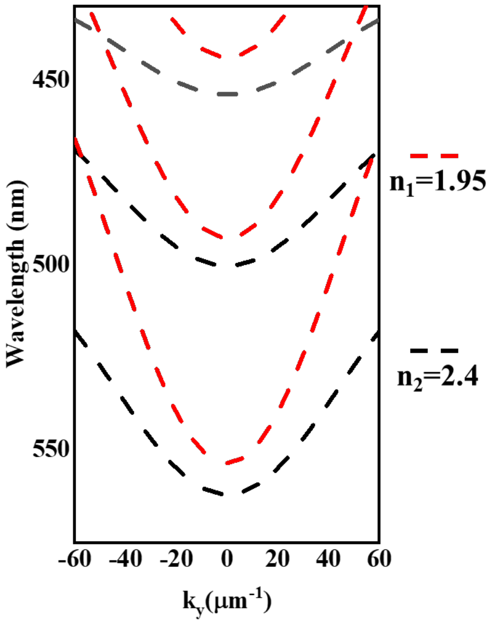

Figure S9. The corresponding refractive index of the two simulated cavity modes (i.e., Y-polarized mode and X-polarized mode).

We have calculated X- and Y-polarized modes by using the two-dimensional cavity photon dispersion relations. According to the equation,

$$E_{\mathrm{CMn}}(\theta) = \sqrt{\left(E_c^2 \times \left(1 - \frac{\sin^2\theta}{n_{\mathrm{eff}}^2}\right)^{-1}\right)} - (n-1) \times l$$

Where θ represents the incidence angle, $E_{\mathrm{CMn}}(\theta)$ is the cavity photon energy of the nth cavity mode as a function of θ, E_{c} represents the cavity modes energy at $\theta = 0°$, $E_{CM1}(\theta)$ represents the energy of the first cavity mode when $n = 1$, $(n-1) \times l$ represents the energy difference from the first cavity mode. The refractive indices are calculated to be 1.95 and 2.40 for the red and black curves, respectively.

OLED array of 6M-DSB crystals

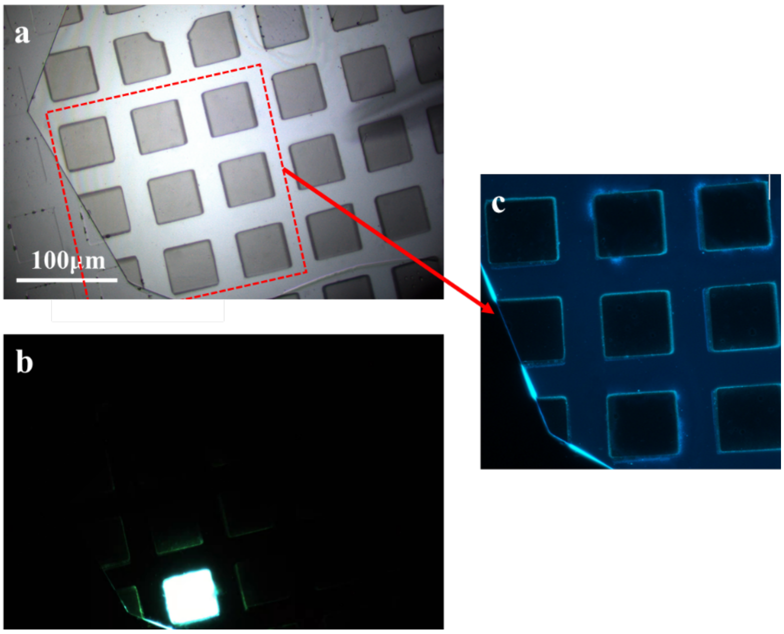

Figure S10. OLED array and the corresponding electroluminescent photographs and the microfluorescence image. (a) Microscopic bright field image of 6M-DSB crystal OLED array. (b) Electroluminescent photographs of individual devices. Under electroluminescence, the device exhibits uniformly bright electroluminescence and edge waveguide. (c) Microfluorescence image of the red box area in (a).

Angular resolved spectra of the device

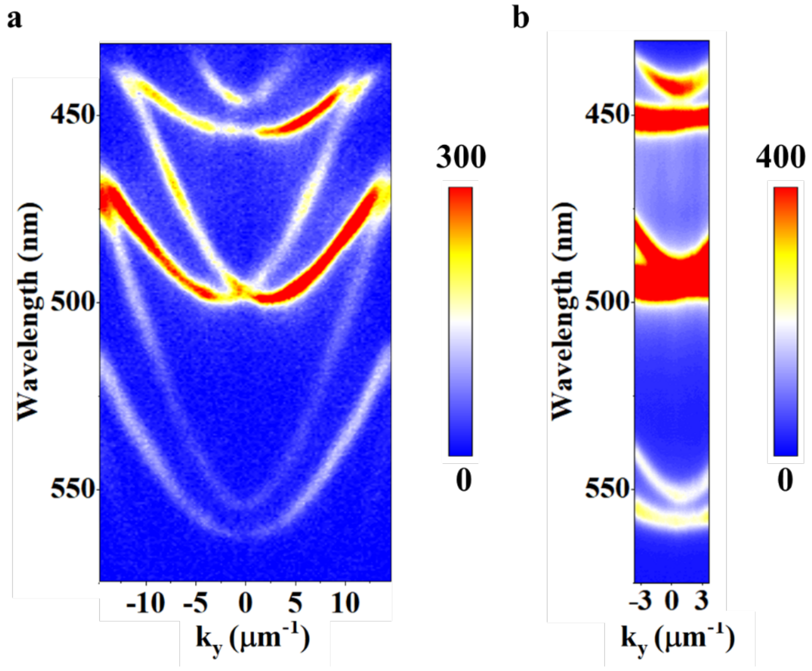

Figure S11. Angle-resolved spectra. (a) Angle-resolved PL spectra under 405-nm laser excitation. (b) Angle-resolved EL spectra of the same device.

Angular resolved spectra of the devices with the organic-crystal thickness of 835 nm, 990nm and 1325 nm.

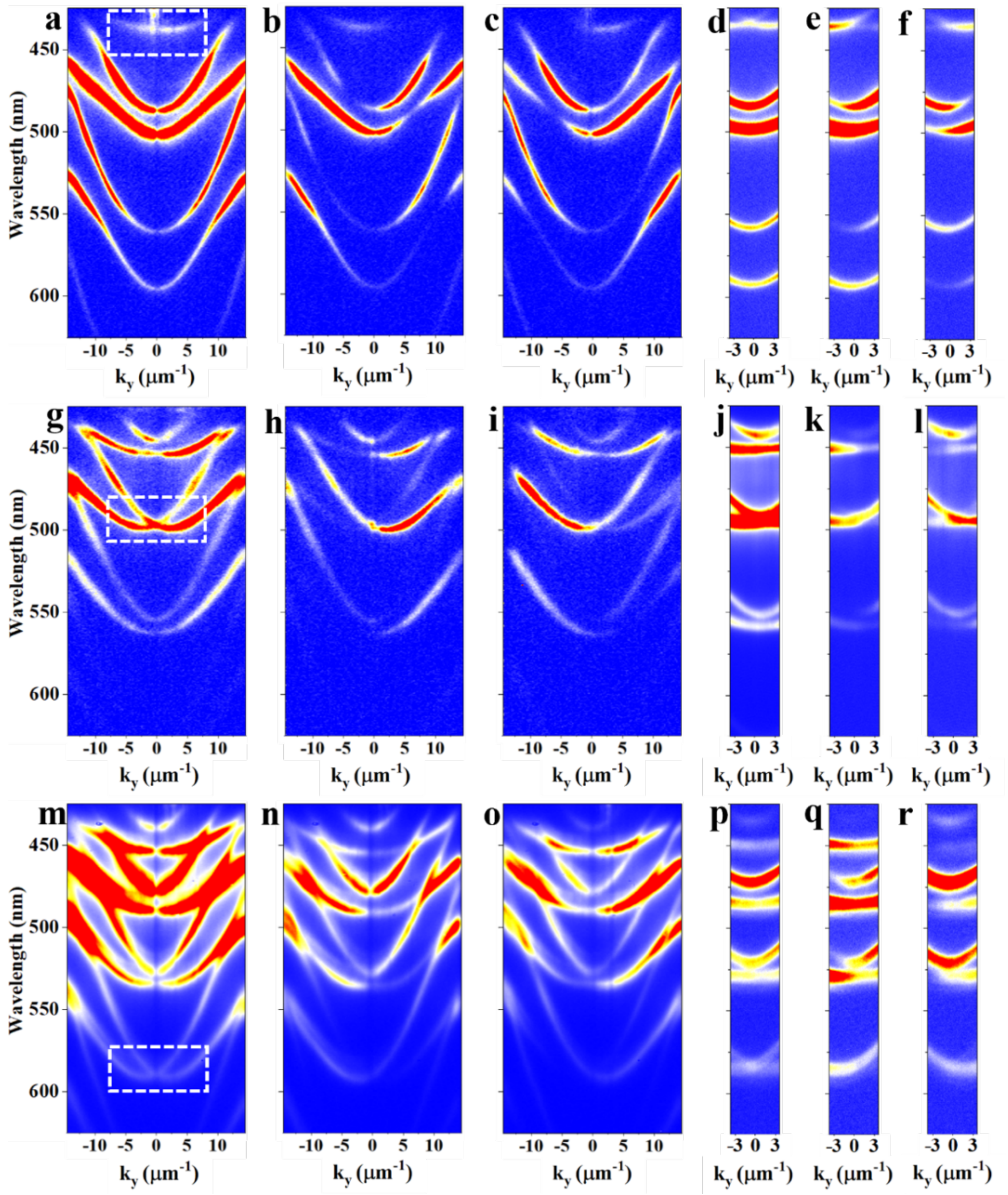

Figure S12. Angle-resolved spectra at different crystal thickness. Angle-resolved photoluminescence (a,g,m) and electroluminescence spectra (d,j,p) of the organic-crystal microcavity with the crystal thickness of 835 nm, 990 nm and 1325 nm, respectively. The white dashed rectangles mark the positions where the RD effect occurs. The corresponding circularly polarized angle-resolved spectra of PL (b,c) and EL (e,f) with the organic-crystal thickness of 835 nm, PL (h,i) and EL (k,l) with the organic-crystal thickness of 990 nm, and PL (n,o) and EL (q,r) with the organic-crystal thickness of 1325 nm.

Table S1. Summary of PL and EL Performance of the Devices with Different Thickness.

Thickness (nm)	V_{on} (V)	Current density (A/m^2)	Luminous (cd/m^2)	EQE (%)	g_{EL}	g_{PL}
835	19	36.77	60829	0.84	$-1.20 \leq g_{EL} \leq 1.14$	$-1.27 \leq g_{PL} \leq 1.42$
990	20	22.7	59324	0.96	$-1.25 \leq g_{EL} \leq 1.11$	$-1.30 \leq g_{PL} \leq 1.43$
1325	31	10.01	53868	1.008	$-1.23 \leq g_{EL} \leq 1.07$	$-1.31 \leq g_{PL} \leq 1.40$

In order to investigate the relation between the thickness of 6M-DSB crystal and the dissymmetry factor for circularly polarized emission, we chose three typical 6M-DSB crystals with the thickness of 835 nm, 990 nm and 1325 nm and performed their angle-resolved photoluminescence and electroluminescence spectra. As shown in Figure S12, the location of the crossing point, characteristic feature of the RD effect, shifts from shorter wavelength to longer wavelength as the increase of the crystal thickness. We calculated the dissymmetry factors of these three devices and the results are summarized in Table S1. The photoinduced and electroinduced dissymmetry factors are almost the same as those of 990-nm devices in the manuscript. Therefore, it can be concluded that the crystal thickness affects only the position of the crossing point induced by the RD spin-splitting not the dissymmetry factor.

Reflection Spectrum and Refractive Index of 6M-DSB Single Crystal

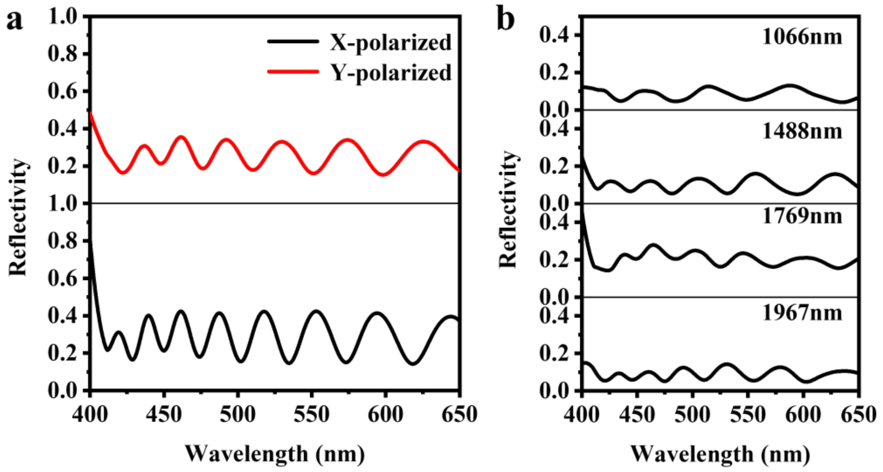

Figure S13. Reflection spectra of different crystal thickness. (a) Reflection spectra of the 2126-nm-microbelt cavity at X-polarization (black line) and Y-polarization (red line) (b) X-polarized reflection spectra of organic cavities with thickness of 1066 nm, 1488 nm, 1769 nm and 1967 nm.

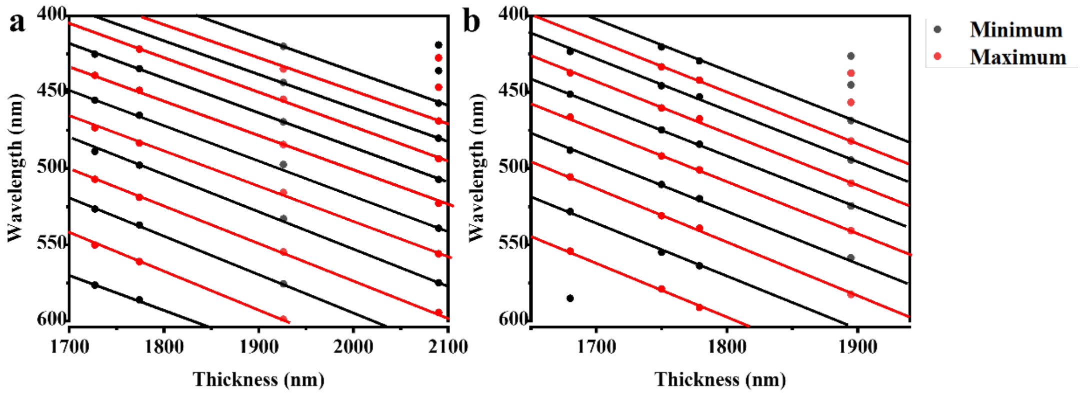

Figure S14. Wavelength of the interference maximum (red points) and minimum (black points) observed in reflection spectra of X-polarization (a) and Y-polarization (b).

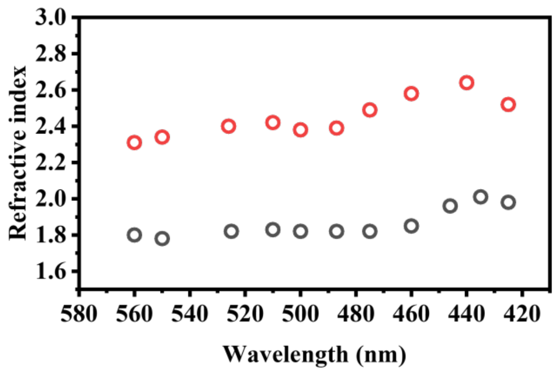

Figure S15. The calculated refractive index of 6MDSB microbelts in X-direction (black dot) and Y-direction (red dot).

We measured the refractive index using the method in the reference[3]. Firstly, the 6M-DSB microbelts are placed on the quartz substrate, and the Fabry-Pérot interference is formed on the top and bottom smooth surfaces. The reflection spectra of the samples are measured with polarized light on parallel to and perpendicular to the long direction of the crystal. Figure S13 shows the reflection spectrum of the sample with the thickness of 2126 nm, where the red line and black line represent the reflection spectra parallel to and perpendicular to the long direction of the crystal, respectively. The interference peak spacing in X- and Y-direction does not change significantly.

Figure S13b shows X-polarized reflection spectra of organic cavities with thickness of 1066 nm, 1488 nm, 1769 nm and 1967 nm, respectively. The interference conditions are given by $2n(\lambda)d = m\lambda$, where $n(\lambda)$ is the refractive index at wavelength λ, m is the order of interference, and d is the crystal thickness. In the reflection spectra, the interference minimum occurs when m is an integer and the maximum occurs when m is a half integer. The wavelengths of the interference maximum and minimum were extracted from the reflection spectra (Figure S13) measured when the polarization of incident light parallel to and vertical to the long direction of microbelts and are plotted as a function of crystal thickness (Figure S14a). The black points correspond to the minimum and the red points correspond to the maximum. The black (red) lines are

fitted by the black (red) points. Since two adjacent black (or red) lines have difference 1 in the interference order, the order m can be determined by $m = d_1/(d_2-d_1)$, where d_1 and d_2 ($d_2 > d_1$) are the thicknesses corresponding to the order m and m+1, respectively, at a fixed wavelength λ. The calculated $n(\lambda)$ is shown in Figure S15. The $n(\lambda)$ in the direction parallel to the long direction of the microbelt rises from 2.31 at 560 nm to 2.52 at 425 nm, and rises from 1.80 at 560 nm to 1.98 at 425 nm in vertical direction (Figure S15). This is basically consistent with our simulation results.

References

1. Gao F, Liao Q, Xu ZZ, Yue YH, Wang Q, Zhang HL, Fu HB, Strong Two-Photon Excited Fluorescence and Stimulated Emission from an Organic Single Crystal of an Oligo(Phenylene Vinylene). *Angew. Chem. Int. Ed.* 2010, 49: 732-735.
2. Xu ZZ, Liao Q, Shi Q, Zhang HL, Yao JN, Fu HB, Low-Threshold Nanolasers Based on Slab-Nanocrystals of H-Aggregated Organic Semiconductors. *Adv. Mater.* 2012, 24, OP216-OP220.
3. Hashimoto S, Ohno N, Itoh M, Exciton-Polariton Dispersion of Thin Anthracene Crystals in the Thickness Range of 3 to 0.1 μm. *Phys. Status. Solidi. (b)* 1991, 165, 277-286.
